# Supplementary material for: Expectations and Experiences of Patients Recently Initiated to Centre-Based Dialysis Treatment
Source: Healthcare (Basel). 2022 May 12;10(5):897. doi: 10.3390/healthcare10050897 (PMC9141855; doi:10.3390/healthcare10050897)
Supplement: Supplementary file 1 [file healthcare-10-00897-s001.zip › healthcare-1681658-supplementary.pdf]

Table S1. Interview guide used in a study exploring the experiences and expectations of patients recently initiated into centre-based dialysis.

**Interview guide**	**Rationale**	**Questions**
Introduction:<br><br>Presentation of interviewer and aims of the study	Who am I? Who is the observer?	I am a master's medical student from the University of Southern Jutland and am involved in a research project from the Hospital of Soenderjylland. The project is about dialysis patients; their experiences and expectations regarding dialysis treatment.<br>My observer is…
	Aim of the focus group discussion—important not to influence or affect participants.	We're here today to learn from your experiences—both positive and negative experiences are very important so that we can gain new insights into this area in order to improve and adjust our offer to dialysis patients.
About the interview	Form of the interview—encourage dynamics in the group between participants but with an awareness of oneself or researcher as a co-constructer.	A focus group discussion is little different from an ordinary conversation; the conversation between you, as a group, is most important. I'll ask you some questions and ask you to discuss them among yourselves. There are no right or wrong answers; all experiences are important. I would like all of you to get involved. You can build on each other's statements or pick up a thread from one another, but please don't interrupt one another, as it will be difficult for me to transcribe afterward.
	Practical information about recording and anonymity.	I'm recording the conversation and I'll anonymise it so that no one can recognise you afterward. When I'm finished writing up the transcription, we will delete the recording. All of this information is also written in the letters of consent that you signed.
	Encouragement of in-group anonymity.	Therefore, I will ask all of you to respect each other's confidentiality—everything that we say in here stays in here and information about who is here will not be shared.
	Timeframe—so that participants know what to expect.	The conversation will last about an hour. Does anybody need anything to drink or use the bathroom before we start?

	Who does what—to explain our roles in relation to moderator and observer?	I will start by asking a question to open the discussion. I may also ask for a further explanation once in a while or ask other questions. My colleague over there will observe and take note of how the conversation is going.
	Name signs if there are a lot of participants who don't know each other beforehand, to make it more personal for the participants and easier for the moderator.	To make things easier, can I please ask you to write your first name on the sign in front of you and place it before you?
Research question:<br>To investigate patients' positive and negative thoughts, experiences, and expectations of their dialysis.	Questions have to concern:<br>- Effect of dialysis<br>- Expectations for dialysis	
Important to get all of the participants to join in and hear their voices so that they can be told apart from each other.	Ice-breaker	Name round<br><br>Have you previously had dialysis together?
A broad question to open up the conversation<br>Invite participants to share both good and bad experiences and get different views on the topic.	Effect of dialysis<br><br>In interpretive description, variation of data is important. (Lomborg)	How has dialysis affected you on an everyday basis?<br><br>• What were your thoughts in relation to everyday work?<br><br>• What have your families said?
A new question that takes a step back and allows participants to think about their initial expectations of dialysis, followed by a question that encourages participants to compare their answers from the previous question to this question.	Expectations of dialysis	What were your expectations before you started dialysis?<br><br>• Have your experiences lived up to your expectations?
Closing question:	Ask participants if they would have chosen differently. An indirect question about regretting their choice in relation to dialysis.	If you were to choose again, would you have chosen the same way, and why?<br><br>• What is your advice to others starting dialysis?

<table>
<tr><td>Only if time allows.</td><td>Close the conversation with an opportunity for participants to offer their advice and suggestions for improvements. (Malterud).
The interview guide is built up with a natural progression in the questions.</td><td>Do you have any suggestions for improvement in relation to starting dialysis?</td></tr>
<tr><td>Supplementary questions

(only if necessary)

An exercise to promote discussion

Each participant gets 3 pre-printed cards with 3 possibilities which they have to rank from 1 – 3 where 1 is what they would prefer most. The most preferred possibility will then be discussed further in the group.</td><td>Ask about the following, if it does not come out in the discussion.

A 2-minute exercise on post-its.

Post-its, if used, may be entered as data (Bente Halkjær).</td><td>Which of the following would you prefer?
Home dialysis
Dialysis at the hospital
A mixture of both
Why?</td></tr>
</table>
